# Supplementary figures and images for: S100A4-lineage cells contribute modestly to angiotensin II-mediated thoracic aortic aneurysms through angiotensin II type 1a receptor in mice
Source: PLoS One. 2026 May 12;21(5):e0348111. doi: 10.1371/journal.pone.0348111 (PMC13166914; doi:10.1371/journal.pone.0348111)

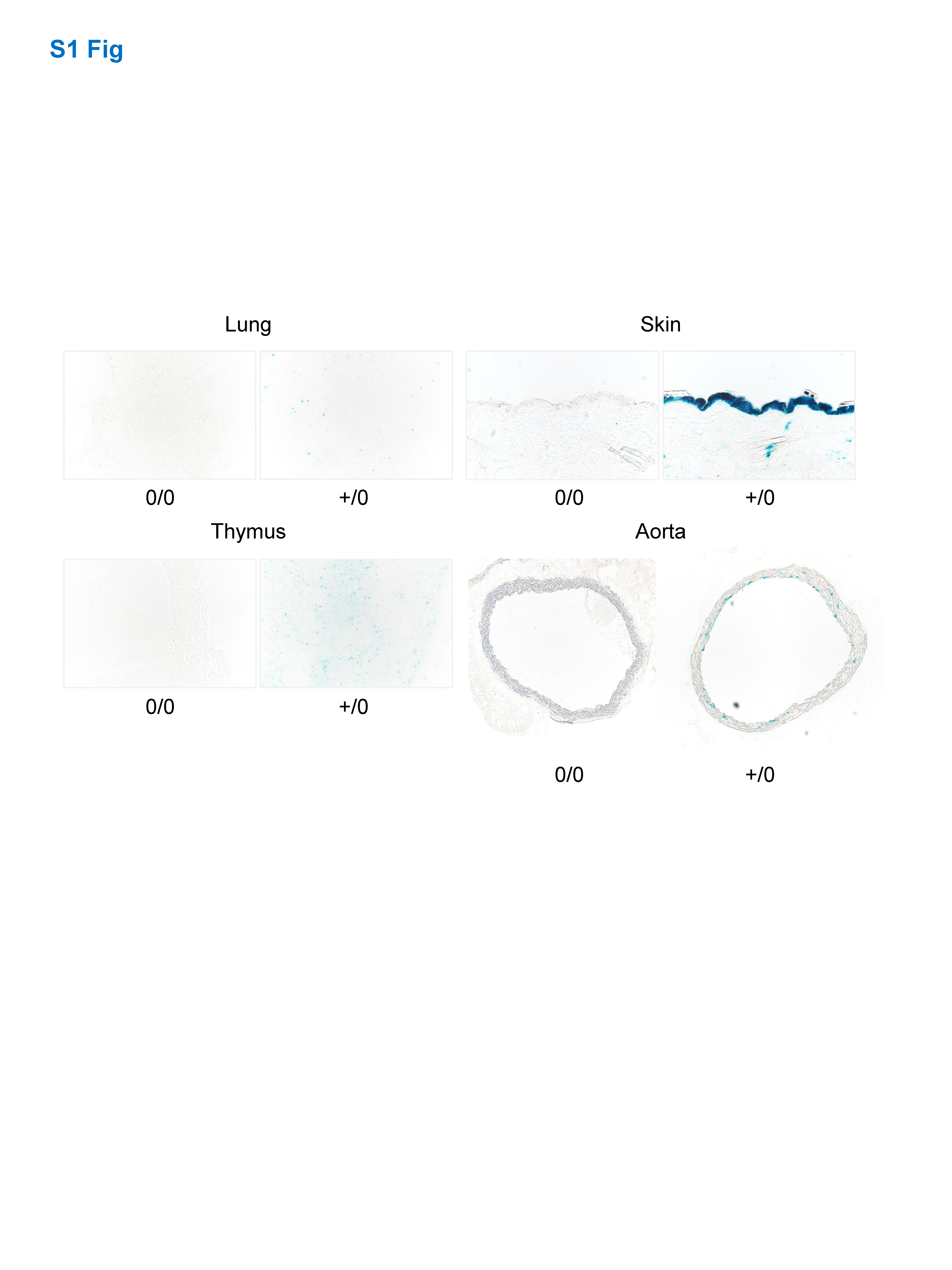

Supplement: S1 Fig — Representative histological images of β-gal staining in the lung, skin, thymus, and aorta of male S100A4-Cre 0/0 (left) and +/0 (right) ROSA26RLacZ + /0 mice. (TIF) [file pone.0348111.s001.tif]

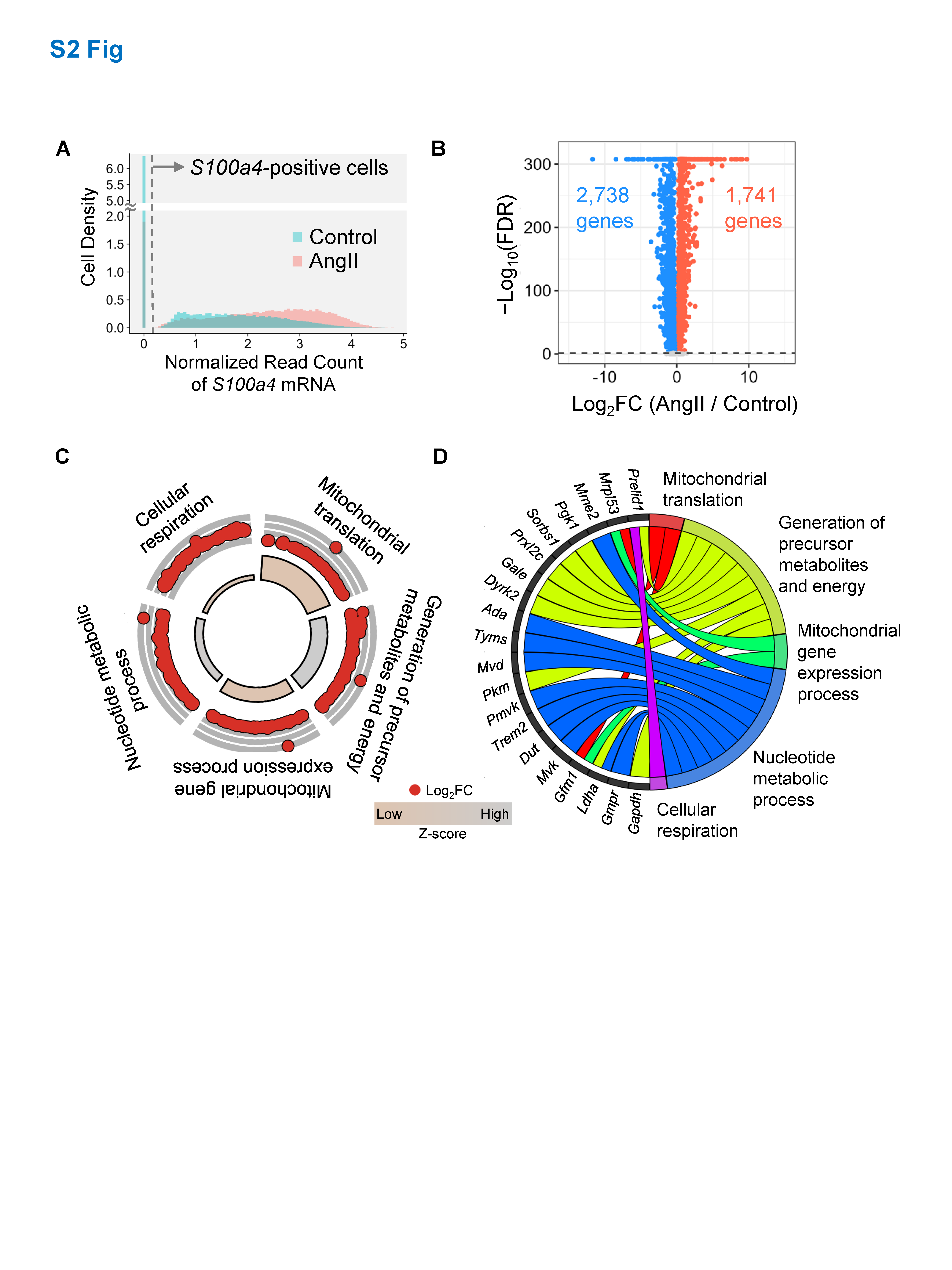

Supplement: S2 Fig — (A) Identification of S100a4-positive cells based on S100a4 mRNA read counts. (B) Volcano plot, (C) gene ontology (GO) enrichment circle plot, and (D) GO enrichment chord plot generated from differentially expressed genes in S100a4-positive cells of the ascending aorta between control and AngII-infused mice. (TIF) [file pone.0348111.s002.tif]
