# Supplementary material for: S100A4-lineage cells contribute modestly to angiotensin II-mediated thoracic aortic aneurysms through angiotensin II type 1a receptor in mice
Source: PLoS One. 2026 May 12;21(5):e0348111. doi: 10.1371/journal.pone.0348111 (PMC13166914; doi:10.1371/journal.pone.0348111)
Supplement: Attachment — Submitted filename: R2R 2nd - S100A4 1-15-26.docx [file pone.0348111.s005.docx]

**Response to Reviewers’ Comments**

**Manuscript ID:** PONE-D-25-61048

**Title:** S100A4-lineage Cells Contribute Modestly to Angiotensin II-mediated Thoracic Aortic Aneurysms Through Angiotensin II Type 1a Receptor in Mice

We thank the Reviewers for their constructive comments and suggestions to improve this manuscript. The Reviewers’ comments are in *italic,* followed by our point-by-point responses. Changes in the revised manuscript are distinguished in blue. We hope that this version has successfully responded to the Reviewers concerns that have assisted in substantially improving the scientific quality of the revision.

**Reviewer 1**

1. *The absence of β-gal staining tissue sections in Fig 2. β-gal staining is prone to non-specific staining, and sections from various organs need to be prepared to determine the specific location of staining.*

We appreciate Reviewer 1’s comments. To follow Reviewer 1’s suggestion, representative images of β-gal–stained sections have been added as **new S1 Fig**. In the staining, counterstaining was not performed to enhance the visibility of β-gal signals. Therefore, it is difficult to determine the precise localization in the lung and thymus. However, no β-gal positive area was observed in any tissues from S100A4-Cre 0/0; ROSA26R^LacZ^ +/0 control mice, supporting the notion that signals observed in S100A4-Cre +/0 mice are specific for S100A4-lineage cells.

Lung

Skin

Thymus

Aorta

0/0

+/0

0/0

+/0

0/0

+/0

0/0

+/0

**S1 Fig. Distribution of S100A4-lineage cells in fibrous organs.** Representative histological images of β-gal staining in the lung, skin, thymus, and aorta of male S100A4-Cre 0/0 (left) and +/0 (right) ROSA26R^LacZ^ +/0 mice.

Line 265,

Histological analyses further validated that β-gal staining was detected only in S100A4-Cre +/0 mice, but not in S100A4-Cre 0/0 controls, supporting the specificity of the staining (S1 Fig).

2. *In lines 272 and 273, the author describes that "β-gal staining was performed in the aorta from S100A4-Cre 0/0 or +/0 ROSA26RLacZ +/0 mice infused with either saline or AngII for 28 days." According to the author's description, there should be four groups: 1. Saline-S100A4-Cre 0/0, ROSA26RLacZ +/0 2. Saline-S100A4-Cre +/0, ROSA26RLacZ +/0 3. AngII-S100A4-Cre 0/0, ROSA26RLacZ +/0 4. AngII-S100A4-Cre +/0, ROSA26RLacZ +/0. However, it is confusing that only two groups are shown in Fig.*

We apologize for the confusing description. As shown in the new S1 Fig, no β-gal–positive areas were detected in aortas of S100A4-Cre 0/0 mice. Therefore, subsequent analyses were focused on S100A4-Cre +/0 mice. In Fig. 3, we compared saline- and AngII-infused S100A4-Cre +/0 mice to determine whether, and how, AngII changes in S100A4-lineage cell distribution. We have revised the manuscript to provide a clear description.

Line 276,

β-gal staining was performed in the aorta from S100A4-Cre +/0 ROSA26RLacZ +/0 mice infused with either saline or AngII for 28 days.

3. *The knockout efficiency of S100A4-AT1aR -/- mice has not been validated, and no corresponding data is provided in the article.*

Thank you for the important suggestions. As shown in the new S1 Fig, the baseline population of S100A4-lineage cells in the aorta is small. Therefore, it is difficult to validate the deletion of AT1aR in S100A4-lineage cells by bulk assays, such as qPCR and Western blotting analyses, using the whole aortas. In addition, there is compelling evidence that antibodies are unable to detect AT1aR protein in either Western blotting or immunostaining (PMID: 22843099, 23150519, 21252156). Therefore, we were not able to perform immunostaining for AT1aR protein to validate its deletion in aortic S100A4-lineage cells. In the present study, we validated its deletion by the presence of Cre and flox sequences in DNA PCR using tail tissues (**Figure for the Editor and Reviewers**). We also validated S100A4-Cre–mediated recombination by LacZ expression using the ROSA26 reporter system. It is worth noting that multiple studies (PMID: 21252156, 23236507, 26160957) have used the same *Agtr1a* floxed mouse line for cell type-specific AT1aR deletion. Based on these data and literature evidence, we concluded that AT1aR was deleted in S100A4-lineage cells in S100A4-Cre AT1aR floxed mice.

**Figure for the Editor and Reviewers**

Representative images of DNA PCR for AT1aR flox and Cre using tails of S100A4-Cre 0/0 and +/0 mice. IL2 was used as an internal control for DNA PCR of Cre.

350 bp

179 bp

222 bp

Wild/Wild Type

AT1aR flox/flox

350 bp

222 bp

Cre 0/0

Cre +/0

Cre

IL-2

4. *The article does not mention the incidence rate of AngII-induced TAA in mice. According to previous literature reports, AngII mainly induces abdominal aortic aneurysms, and the incidence rate of TAA is relatively low.*

Previous reports by many groups and ours have shown that AngII infusion induces a highly reproducible dilation of the ascending aorta, with significant expansion observed in nearly all infused mice (PMID: 25038458, 26891740, 35770665). Importantly, AngII-induced TAA develops as a continuous spectrum of severity, and is typically ~0.5 mm, rather than binary phenotypes of AngII-induced AAA (PMID: 25038458, 35045728). Therefore, reporting the incidence rates using a fixed cutoff for TAA in this model may result in an underestimation. In the present study, we assessed disease severity by measuring the area of the thoracic aorta, which has been used in previous studies (PMID: 20088827, 25038458, 33693132) for reliable evaluation of TAA severity.

5. *The data in Data Summary-S100A4 PO.xlsx is also confusing. What does SBP=26, 55, 32 mean?*

This was a labeling error, and it has been corrected in the revised Excel file.

**Reviewer 2**

1. *The conclusion section of the abstract repeats the term "modestly." It is suggested to replace it with synonyms such as "partially" or "to a limited extent" to enhance linguistic diversity.*

Thank you for the suggestion. We have replaced “modestly” with “partially”.

Line 33,

Deletion of AT1aR in S100A4-lineage cells partially reduced AngII-induced TAA formation.

2. *The background introduction is comprehensive but could briefly mention the known roles of S100A4 in vascular diseases to strengthen logical coherence.*

S100A4 promotes atherosclerosis by promoting SMC phenotypic switching and amplifying inflammatory responses (PMID: 32914661, 33135065). We have added this information on the roles of S100A4 in the Introduction section of the revised manuscript.

Line 63,

In addition to its role in organ fibrosis, S100A4 is involved in atherosclerosis in mice and humans through promoting SMC phenotypic switching and amplifying inflammatory responses.[14, 15]

3. *The description of the animal models is clear, but it is recommended to supplement the strain source of the S100A4-Cre mice and provide citations for specificity validation. The authors need to provide or cite published literature to demonstrate that the "S100A4-Cre mouse" tool they used can specifically and reliably label the cell population they intend to study (i.e., S100A4-lineage cells) in practical applications.*

In accordance with the Reviewer’s suggestion, the source of the S100A4-Cre mice (The Jackson Laboratory: #012641) has been described in the Methods section. We have also added citations demonstrating that the S100A4-Cre line has been validated and is widely used as a reliable tool to label S100A4-lineage cells in cardiovascular studies (PMID: 14764882, 23997102, 26160957).

Line 84,

C57BL/6J (#000664), S100A4-Cre (#012641),[16-18] ROSA26RLacZ (#003474), and low-density lipoprotein receptor-deficient (LDLR-/-, #002077) mice were purchased from The Jackson Laboratory.

4. *Figure 3D: The co-staining images are very convincing. It is suggested to use arrows in the figure to clearly indicate typical co-localized cells for easier reader interpretation. Additionally, increasing the image resolution would be beneficial.*

We thank the Reviewer for this suggestion. We have replaced the images with improved resolution and added arrows to Figure 3D to indicate representative co-localized cells.

β-gal / α-SMA

Saline

AngII

Lumen

Lumen

**Fig 3**

β-gal / ER-TR7

Saline

AngII

Lumen

Lumen

Line 293,

Black arrows indicate representative β-gal positive cells co-localizing with α-SMA or ER-TR7 in the aortic wall.

5. *The scRNA-seq analysis pipeline is detailed, but a brief explanation of the thresholds used for cell clustering and marker gene selection could be added.*

We have expanded the Method section to describe the thresholds used for cell clustering and marker genes.

Line 200,

Uniform manifold approximation and projection (UMAP) dimensionality reduction using the first 20 principal components (PCs) was applied, and cell clustering was performed using a shared nearest neighbor approach with a resolution parameter of 0.5. “FindAllMarkers” and “FindConcervedMarkers” functions were used to identify conserved marker genes to determine cell types of each of the clusters. Cell clusters were annotated based on canonical lineage markers. Smooth muscle cells were identified by expression of *Myh11*, *Acta2*, and *Tagln*, endothelial cells by *Pecam1* and *Cdh5*, fibroblasts by *Dcn*, *Lum*, and *Col1a1*, and macrophages by *Lyz2* and *Cd68*. These marker genes were used to define the major cell populations in UMAP projections and downstream analyses.

6. *Statistical Analysis Section: It is recommended to explicitly state whether multiple comparison corrections were performed (e.g., in addition to Holm-Sidak).*

We have clarified in the Statistical Analysis section that multiple comparison correction was performed using the Holm–Sidak method for post hoc analyses following ANOVA.

Line 184,

TMM-normalized read count, protein intensity data, and number of β-gal positive cells were analyzed by Student’s t-test. For datasets involving four groups, two-way ANOVA followed by Holm–Sidak multiple comparison correction was applied.

7. *It is suggested to further discuss the specific molecular mechanisms through which S100A4 cells influence TAA via the AT1aR signaling pathway (e.g., fibrosis, inflammation, SMC phenotypic switching).*

As shown in S2 Fig, gene ontology analysis of S100A4-positive cells revealed significant enrichment of mitochondrial-related pathways following AngII infusion, suggesting alterations in mitochondrial function in these cells. Emerging evidence indicates that mitochondrial dysfunction plays an important role in aortic aneurysm pathogenesis (PMID: 33709773, 39945066, 40277943). We have incorporated these points and relevant literature into the revised Discussion to provide a mechanistic framework, while acknowledging that direct validation of these pathways will require future studies.

Line 361,

Our scRNAseq analysis revealed that AngII infusion significantly altered mitochondrial-related mRNA abundance in S100a4-positive cells. These findings suggest that AngII modulates mitochondrial function and metabolic regulation in S100a4-positive cells. Increasing evidence indicates that mitochondrial dysfunction contributes to aortic aneurysm development.[37-39] Thus, it is plausible that mitochondria-associated pathways in S100A4-lineage cells may contribute to the modest effects observed in the present study. Further investigation is needed to elucidate the molecular mechanisms and cellular interactions through which S100A4-lineage cells mediate these pathologies.

8. *The formatting of some references is inconsistent (e.g., mixed use of journal name abbreviations and full names).*

All references have been revised to follow the journal guidelines.
