# Supplementary material for: S100A4-lineage cells contribute modestly to angiotensin II-mediated thoracic aortic aneurysms through angiotensin II type 1a receptor in mice
Source: PLoS One. 2026 May 12;21(5):e0348111. doi: 10.1371/journal.pone.0348111 (PMC13166914; doi:10.1371/journal.pone.0348111)
Supplement: Attachment — Submitted filename: R2R - 3-2-26 hs.docx [file pone.0348111.s006.docx]

**Response to Reviewers’ Comments**

**Manuscript ID:** PONE-D-25-61048R1

**Title:** S100A4-lineage Cells Contribute Modestly to Angiotensin II-mediated Thoracic Aortic Aneurysms Through Angiotensin II Type 1a Receptor in Mice

We thank the Reviewer for the suggestions to improve this manuscript. The Reviewer’s comments are in *italic,* followed by our point-by-point responses. Changes in the revised manuscript are distinguished in blue. We hope that this version has successfully responded to the Reviewer’s concerns.

**Reviewer 2**

1. *The limitation in validating AT1aR knockout should be more explicitly stated.
   In their response, the authors explained that due to the lack of reliable antibodies and the low baseline abundance of S100A4-lineage cells, direct validation of AT1aR deletion in aortic S100A4-lineage cells was not feasible. This is an honest scientific limitation. Although this was clarified in the response letter, a brief statement should be added to the main text, particularly in the Results section describing Figure 5. For example, the authors could add: Due to the lack of reliable AT1aR antibodies and the low basal abundance of S100A4-lineage cells in the aorta, we were unable to directly validate AT1aR deletion efficiency in these cells by immunostaining or Western blotting. However, the presence of Cre and floxed alleles was confirmed by DNA PCR using tail tissue (data not shown), and S100A4-Cre-mediated recombination activity was validated using the ROSA26 reporter system (Figure 2), supporting the effectiveness of the genetic knockout model.*

We appreciate the constructive comments. The manuscript has been revised to follow Reviewer #2’s suggestions.

Line 307,

Due to the lack of authenticated antibodies to detect AT1aR protein and the low basal abundance of S100A4-lineage cells in the aorta, it was not feasible to directly validate the efficiency of AT1aR deletion in these cells by either immunostaining or Western blotting. However, the presence of Cre and floxed alleles was confirmed by DNA PCR using tail tissues (data not shown), and S100A4-Cre-mediated recombination activity was validated using the ROSA26RLacZ reporter system (**Fig 2**) to support the effectiveness of AT1aR deletion.

2. *The description of experimental groups in Figure 3 should be more precise.
   In the original manuscript, Figure 3 only shows results from S100A4-Cre-positive mice infused with either AngII or saline. Since Cre-negative mice showed no LacZ signal at all, it is reasonable to compare only the positive groups.
   To avoid any confusion for readers, it is recommended to explicitly state this in the legend of Figure 3. For example: No β-gal signal was detected in S100A4-Cre-negative control mice (see S1 Fig); therefore, only S100A4-Cre-positive mice are shown and compared here following either saline or AngII infusion.*

The figure legend of Fig 3 has been revised to follow Reviewer #2’s suggestion.

Line 293,

Since no β-gal signal was detected in S100A4-Cre-negative control mice (S1 Fig), only S100A4-Cre-positive mice are shown and compared following either saline or AngII infusion.

3. *Terminology and formatting should be consistent.
   Several instances of "Angll" (using the letter 'l' instead of the Roman numeral 'II') remain in the manuscript, particularly in the "Response to Reviewers" section.
   Please carefully review the entire manuscript (including the main text, figures, supplementary materials, and the response letter) and correct all instances of "Angll" to "AngII."*

We have carefully reviewed the entire manuscript, including the main text, figures, supplementary materials, and the response letter, to ensure that the Roman numeral “II” is consistently used instead of the lowercase letter “ll.”
