# Supplementary material for: S100A4-lineage cells contribute modestly to angiotensin II-mediated thoracic aortic aneurysms through angiotensin II type 1a receptor in mice
Source: PLoS One. 2026 May 12;21(5):e0348111. doi: 10.1371/journal.pone.0348111 (PMC13166914; doi:10.1371/journal.pone.0348111)
Supplement: Attachment — Submitted filename: Response to Reviewers 4-7-26.docx [file pone.0348111.s007.docx]

**Response to Editors’ Comments**

**Manuscript ID:** PONE-D-25-61048R2

**Title:** S100A4-lineage Cells Contribute Modestly to Angiotensin II-mediated Thoracic Aortic Aneurysms Through Angiotensin II Type 1a Receptor in Mice

We thank the Editor for the suggestions to improve this manuscript. The Editor’s comments are in *italic,* followed by our point-by-point responses. Changes in the revised manuscript are distinguished in blue. We hope that this version has successfully responded to the Editor’s concerns.

**Editor**

1. *Direct Validation of the AT1aR Knockout: An Explicit and Unambiguous Statement of a Critical Limitation*

*Problem: The study lacks direct biochemical evidence (e.g., Western blot, qPCR on sorted cells) proving that the deletion of Agtr1a (AT1aR) in the target S100A4-lineage cells was successful and efficient. While ROSA26 reporter validation (Fig. 2) confirms Cre recombinase activity, it does not confirm the functional knockout of the floxed Agtr1a gene. This is a major methodological weakness that introduces significant uncertainty into the interpretation of the observed phenotype. The causal link between the genetic manipulation and the modest reduction in TAA is therefore inferred, not directly proven.*

*Required Action: The authors must provide an unequivocal and prominently placed statement in the Results section (and note in Methods) detailing this impossibility. Placement: This statement must be added immediately following the description of the mouse model and the presentation of the TAA results (Fig. 5), ideally as a dedicated paragraph.*

*Content: The statement should:*

1. *Explicitly state that direct validation of the AT1aR knockout at the protein or mRNA level in the specific S100A4-lineage cell population was not technically feasible.*
2. *Clearly articulate the dual technical barrier: (a) the complete lack of reliable, specific antibodies for mouse AT1aR, and (b) the prohibitively low abundance of S100A4-lineage cells in the aorta, which makes biochemical isolation (e.g., by FACS) for qPCR or Western blot unachievable with current technology.*
3. *Reiterate the supporting evidence that **indicates**, but does not prove, successful knockout: confirmation of all alleles (Cre, floxed AT1aR) by germline DNA PCR, and validation of Cre-mediated recombination activity using the ROSA26-LacZ reporter in the same cellular lineage (Fig. 2, S1 Fig).*
4. *Conclude by stating that the functional knockout is therefore **inferred**, and this limitation must be considered when interpreting the modest phenotype.*

**Authors’ reply:**

We appreciate the Editor’s comments. As suggested by the Editor, we have added a dedicated section following Fig. 5 in the Results to explicitly describe the technical limitations in validating AT1aR deletion in S100A4-lineage cells. We have also added a corresponding statement in the Methods section.

**Changes in the manuscript:**

Line 95,

Due to the lack of antibodies that authentically detect AT1aR, direct validation of protein deletion was not feasible. Recombination efficiency was assessed by lineage tracing.

Line 338,

**Validation of AT1aR deletion in S100A4-lineage cells**

Since no AT1aR antibodies that authentically detect the protein are available, it was not feasible to directly validate the efficiency of AT1aR deletion in these cells by immunostaining or Western blotting. However, the presence of Cre and floxed alleles was confirmed by DNA PCR using tail tissues (data not shown), and S100A4-Cre-mediated recombination activity was validated using the ROSA26R^LacZ^ reporter system (Fig 2), supporting the effectiveness of AT1aR deletion.

Line 366,

Since the efficiency of AT1aR deletion in S100A4-lineage cells could not be directly validated, the modest phenotype could potentially reflect incomplete protein deletion. Nonetheless, these findings suggest that S100A4-lineage cells are not the sole mediators of AT1aR-dependent effects in TAA.

2. *Re-framing the Study's Central Finding: From "Lineage" to "Functional State Marker"*

*Problem: The manuscript's framing, while acknowledging limitations, does not fully grapple with the profound implications of S100A4's expression in multiple cell types (SMCs, fibroblasts, immune cells). The term "S100A4-lineage cells" is used throughout, but the conclusion risks being misinterpreted as defining the role of a single, homogeneous population. The provided literature (e.g., Kong et al., 2013, Österreicher et al., 2011) and the authors' own data (Fig 1, 3) clearly establish S100A4 is not a specific marker.
Required Action: Re-interpret the study's central finding to position S100A4 not as a marker of a specific lineage, but as a **marker of a "disease-activated cellular state"** that is common to multiple cell types undergoing pathological stress. This is the study's most valuable conceptual contribution.
Abstract: Revise the final sentence to explicitly state this heterogeneity and its implications. Example: "In conclusion, S100A4 identifies a heterogeneous population of aortic cells, including smooth muscle cells and fibroblasts, that enter a common disease-associated state. This S100A4-expressing cell population contributes modestly, via AT1aR signaling, to AngII-mediated TAA development in mice, potentially through mechanisms involving mitochondrial reprogramming."
Introduction (Lines 78-80): When introducing the hypothesis, explicitly acknowledge this complexity. Example: "While S100A4 is known to be expressed in multiple mesenchymal and inflammatory cell types under pathological conditions, our previous lineage tracing studies revealed marked expansion of S100A4-expressing cells in the diseased aorta. This prompted us to investigate the functional role of this heterogeneous population, marked by S100A4 expression, as an integrated unit in TAA pathogenesis."
Discussion (Most Important): This is where the most significant changes are required.*

1. *Early and Explicit Acknowledgment: Begin the discussion by contextualizing the findings within the known cellular heterogeneity of S100A4. Example: "It is crucial to interpret our findings within the context of S100A4 as a marker of a disease-activated state rather than a specific cell lineage. Consistent with studies challenging the fibroblast-specificity of S100A4/FSP1, our own data confirm that S100A4-lineage cells in the aorta encompass fibroblasts, a significant population of smooth muscle cells, and potentially other cell types. Therefore, the observed effects of AT1aR deletion reflect the integrated response of this heterogeneous population."*
2. *Formalize the Concept: Introduce a new subsection titled: "S100A4 as a Marker of a Disease-Activated Cellular State and Integrator of Pathological Signals." In this section, synthesize the literature you have provided to build this argument. Discuss how S100A4 expression is upregulated in various cell types (SMCs, fibroblasts, macrophages, etc.) across different diseases (cancer, fibrosis, vascular disease) by common pathological stimuli (TGF-β, hypoxia, inflammation). This positions S100A4 as a convergent node of cellular stress.*

**Authors’ reply:**

We thank the Editor for these important conceptual comments. We completely agree with the editor’s summary that, while S100A4 was thought initially to be a fibroblast-specific gene, it is now known to be expressed in other cell types. This is the rationale for avoiding the previous commonly used terminology of “fibroblast-specific” when describing this Cre. Indeed, moving forward, we are using a nomenclature of the promoter upstream of the Cre, rather than implying it has cell-specificity. After discussion with many colleagues, we concluded that “lineage” would be an optimal term. This is based on the term lineage being defined as descent from an ancestor. We considered the term “driven”, but were concerned that this implies there is continuous active expression. Therefore, we would respectfully submit that “S100A4-lineage cells” is an accurate description of the conditions of this study. However, if this term is contrary to PLoS ONE policy, we would be fully prepared to convert the text to use the term “driven”.

We further acknowledge the Editor’s insightful suggestion that S100A4 may reflect a disease-associated cellular program shared across multiple cell types. While this is an intriguing and potentially important conceptual framework, we respectfully refrain from formally defining S100A4 as a marker of a unified “disease-activated cellular state,” as our current data do not directly demonstrate a common functional phenotype across all S100A4-positive cells. Nevertheless, we have revised the Abstract, Introduction, and Discussion to more clearly emphasize the heterogeneity of S100A4-lineage cells and to avoid any interpretation implying cell type specificity. We believe these changes improve the conceptual clarity and prevent misinterpretation of our findings.

**Changes in the manuscript:**

Line 35,

Following AngII infusion, S100A4-lineage cells expanded markedly throughout the entire aortic wall and comprised a heterogeneous population including smooth muscle cells and fibroblasts.

Line 78,

These findings indicate that S100A4 identifies a heterogeneous population of cells rather than just fibroblasts, as originally defined for this promoter. Accordingly, in this study, we focused on the functional role of this heterogeneous population of S100A4-lineage cells in AngII-induced TAA formation.

Line 350,

These observations indicate that S100A4 identifies a heterogeneous population of cells rather than a single defined lineage.

3. *Deepening the Mechanistic Discussion: Building the "AT1R-S100A4-Mitochondria" Axis*

*Problem: The current discussion (Lines 375-381) merely mentions that "mitochondrial dysfunction may contribute." This is superficial and fails to integrate the study's findings with the rich literature on S100A4's role in mitochondrial regulation (provided in your search).*

*Required Action: Construct a detailed, multi-layered mechanistic discussion that proposes a testable model.*

*Layer 1: Connecting the Transcriptomic Signature to Known S100A4 Functions: Explicitly link the scRNAseq findings (S2 Fig C, D) to the published literature. Example: "Our scRNAseq data revealed that AngII infusion significantly alters the expression of genes related to mitochondrial transcription, translation, and cellular respiration specifically within S100a4-positive cells. This transcriptional signature is strikingly consistent with a growing body of literature demonstrating a direct role for S100A4 in regulating mitochondrial function. For instance, S100A4 has been shown to upregulate the mitochondrial complex I subunit NDUFS2, thereby enhancing oxidative phosphorylation [Liu et al., 2019]. Furthermore, S100A4 is implicated in various mitochondrial-dependent processes, including the regulation of mitophagy [Huang et al., 2026; Lampinen et al., 2022], the response to oxidative stress [Indo et al., 2015], and metabolic reprogramming towards glycolysis [Bettum et al., 2015]."*

*Layer 2: Proposing the "AT1R-S100A4-Mitochondria" Axis: Build an integrated model.
First, discuss the upstream regulation of S100A4. Use the literature you provided to outline potential mechanisms by which AngII/AT1aR signaling could lead to S100A4 upregulation. Example: "The upregulation of S100A4 in response to AngII is likely mediated by AT1aR-dependent activation of downstream signaling pathways. AngII is a well-established activator of TGF-β signaling in vascular cells, and TGF-β has been shown to upregulate S100A4 through Smad-dependent mechanisms. Additionally, AngII can activate MAPK/ERK pathways, which may converge on AP-1 transcription factors known to regulate S100A4. Hypoxia-inducible factors (HIFs) may also contribute, given the presence of hypoxia-responsive elements in the S100A4 promoter. The expression of S100A4 may also be influenced by its epigenetic state, such as DNA methylation of its first intron, which could modulate its inducibility in different cell types or individuals."
Second, connect this to the downstream effects on mitochondria. Example: "Once upregulated, S100A4 appears to act as an intracellular effector, translating these upstream signals into changes in mitochondrial function. By modulating the expression of key mitochondrial proteins like NDUFS2, S100A4 can influence oxidative phosphorylation, cellular ATP levels, and the production of reactive oxygen species. This S100A4-mediated mitochondrial reprogramming may in turn drive pathological processes central to TAA, such as VSMC phenotypic switching, inflammation, and extracellular matrix degradation. " Cite the papers by Oller et al., 2021 and Liu et al., 2025 that you provided earlier.*

*Layer 3: Reinterpreting the "Modest" Phenotype in Light of this Model: Explain why the effect is modest. Example: "The modest contribution of AT1aR deletion in S100A4-lineage cells can now be understood within this integrated model. If S100A4 is a convergent node for multiple upstream pathological signals (TGF-β, hypoxia, etc.), then blocking only one input (AT1aR signaling) would be expected to only partially reduce S100A4 expression and its downstream mitochondrial effects. Other signals could still drive S100A4 upregulation and pathology. Furthermore, the S100A4 lineage itself is heterogeneous; AT1aR signaling may be more critical in some S100A4-expressing cell subtypes (e.g., a specific fibroblast subset) than others, and its deletion in all cells may mask a more significant effect in a particular subpopulation."*

**Authors’ reply:**

We appreciate the Editor’s suggestion. In this study, our single-cell RNA sequencing identified changes in genes related to mitochondrial function in S100a4-lineage cells following AngII infusion. However, we did not perform functional experiments specifically addressing mitochondrial regulation. Therefore, a detailed mechanistic interpretation would be beyond the scope of the present study. We are concerned that providing such text would be viewed by readers as an overinterpretation of the current data. We have expanded the Discussion and incorporated several of the references suggested by the Editor, while avoiding overinterpretation beyond our data.

**Changes in the manuscript:**

Line 393,

This transcriptional signature is consistent with a previous study implicating S100A4 in the regulation of mitochondrial function and cellular metabolism.[43] The previous study has shown that S100A4 upregulates the mitochondrial complex I subunit NDUFS2, thereby enhancing oxidative phosphorylation. In addition, S100A4 has been implicated in multiple mitochondrial-dependent processes, including the regulation of mitophagy, the response to oxidative stress, and metabolic reprogramming.

4. *Proposing Novel and Specific Future Directions
   Problem: The future directions are vague ("further investigation is needed").
   Required Action: Replace generic statements with specific, hypothesis-lineage research questions derived from your new conceptual model.
   On Cell Heterogeneity: "Future studies should employ more specific genetic tools to deconstruct the individual roles of distinct cellular components within the broader S100A4-expressing population. For example, using PDGFRα-CreERT2 to target adventitial fibroblasts or Myh11-CreERT2 to target mature SMCs, combined with AT1aR deletion, could reveal which cell type is the primary driver of the observed phenotype."
   On S100A4 Regulation:"What are the specific transcription factors and cis-regulatory elements responsible for AngII-induced S100A4 expression in aortic cells? Chromatin immunoprecipitation studies targeting Smads, AP-1, and HIF-1α could identify which factors bind to the S100A4 regulatory regions. Furthermore, does the epigenetic state (e.g., DNA methylation) of the S100A4 gene determine cell-type-specific or individual-specific susceptibility to TAA?"
   On Mitochondrial Function: "Is the S100A4-mediated regulation of NDUFS2 and mitochondrial complex I activity necessary for AngII-induced VSMC phenotypic switching or fibroblast activation? Functional studies using S100A4 gain- and loss-of-function approaches in primary aortic SMCs and fibroblasts, combined with Seahorse metabolic flux analysis, are needed to directly test this."
   On Therapeutic Potential: "Can interrupting the pathways upstream of S100A4—such as TGF-β receptor blockade or HIF-1α inhibition—or targeting the S100A4-mitochondria axis directly (e.g., with NDUFS2 inhibitors) attenuate TAA formation more effectively than targeting AT1aR alone?"*

*Summary*

*This revision requires a fundamental shift in the paper's narrative. The core argument should move from:

> "We deleted AT1aR in S100A4-lineage cells and saw a modest effect."

To:
> "We demonstrate that S100A4 identifies a heterogeneous population of cells in a disease-activated state. By integrating our multi-omics data with the extensive literature on S100A4 biology, we propose a novel 'AT1R-S100A4-Mitochondria' axis as a key pathway in TAA. The modest effect of AT1aR deletion, while a limitation of the model, is entirely consistent with S100A4's role as a convergent integrator of multiple pathological signals. This work redefines S100A4 from a disputed lineage marker to a central functional node in vascular pathology."

This reframing turns the study's limitations into part of its strength—a nuanced and honest exploration of a complex system. By building this new conceptual framework, the paper will make a significant and lasting contribution to the field, well beyond the sum of its individual experimental parts.*

**Authors’ reply:**

We appreciate the Editor’s thoughtful suggestions regarding these important future directions. We have included a discussion on the use of more cell-type–specific genetic tools to address the cellular heterogeneity within the S100A4-lineage population. In the revised manuscript, we have also expanded the Discussion to further consider potential regulatory mechanisms of S100A4 expression. In the current revision, we have expanded the Discussion to further address potential regulatory mechanisms of S100A4 expression. We have also elaborated on the potential involvement of mitochondrial-related pathways in S100A4-lineage cells, based on our transcriptomic findings. Regarding therapeutic implications, given that the effect of AT1aR deletion in S100A4-lineage cells was modest, we would posit that it would be too speculative to take the leap of proposing this as a therapeutic strategy. Accordingly, we have limited our discussion to a general perspective without overextending beyond the current data.

We agree that integrating our findings into a broader conceptual framework could enhance the impact of the study. However, we respectfully consider that proposing a defined “AT1R–S100A4–mitochondria” axis would not be sufficiently supported by our current data. The primary finding of this study is that deletion of AT1aR in S100A4-lineage cells modestly attenuates AngII-induced TAA formation. While our transcriptomic analysis suggests potential alterations in mitochondrial-related pathways, we did not perform functional experiments directly addressing mitochondrial regulation. Therefore, establishing a mechanistic axis would go beyond the scope of the present study and could lead readers to assume that the data is being overinterpreted. Accordingly, we have revised the manuscript based on conclusions that are supported by our experimental data.

**Changes in the manuscript:**

Line 356,

We demonstrated that AngII infusion increased S100A4 in the ascending aorta. The transcriptional regulation of S100A4 is linked to multiple signaling pathways and transcription factors, including Smad, Wnt, and hypoxia-inducible factors. AngII-stimulated AT1aR signaling may influence S100A4 expression through these regulatory mechanisms. However, the precise transcriptional and epigenetic control of S100A4 in aortic cells remains to be determined, and further studies are required to clarify these mechanisms.

Line 422,

Thus, future studies should further dissect the roles of distinct fibroblast subsets within S100A4-lineage cells using more cell-type-specific genetic tools, such as PDGFRα-CreERT2 in combination with AT1aR deletion.

5. *Importantly, based on the comprehensive peer review, the following real references have been identified and should be incorporated into the revised manuscript. They are organized thematically to support the new conceptual framework proposed in the review. These citations are not mandatory for the authors to use; rather, they are provided as an optional reference list from the editor’s perspective to assist the authors during the revision process.*

*Complete Reference List for Manuscript Revision*

**Authors’ reply:**

We thank the Editor for providing a comprehensive and thoughtfully curated list of relevant references. We have selectively integrated several key studies into the revised Discussion section where they directly strengthen the interpretation of our findings. In particular, references related to transcriptional regulation of S100A4 and its association with mitochondrial function to further deepen the Discussion. We also cited several studies to further support the lack of a reliable antibody for AT1aR.

**Additional references:**

17.	Iwano M, Plieth D, Danoff TM, Xue C, Okada H, Neilson EG. Evidence that fibroblasts derive from epithelium during tissue fibrosis. J Clin Invest. 2002;110(3):341-50. Epub 2002/08/07. doi: 10.1172/jci15518.

20.	Benicky J, Hafko R, Sanchez-Lemus E, Aguilera G, Saavedra JM. Six commercially available angiotensin II AT1 receptor antibodies are non-specific. Cell Mol Neurobiol. 2012;32(8):1353-65. Epub 2012/07/31. doi: 10.1007/s10571-012-9862-y.

21.	Herrera M, Sparks MA, Alfonso-Pecchio AR, Harrison-Bernard LM, Coffman TM. Lack of specificity of commercial antibodies leads to misidentification of angiotensin type 1 receptor protein. Hypertension. 2013;61(1):253-8. Epub 2012/11/15. doi: 10.1161/hypertensionaha.112.203679.

30.	Wang H, Shi J, Luo Y, Liao Q, Niu Y, Zhang F, et al. LIM and SH3 protein 1 induces TGFβ-mediated epithelial-mesenchymal transition in human colorectal cancer by regulating S100A4 expression. Clin Cancer Res. 2014;20(22):5835-47. Epub 2014/09/26. doi: 10.1158/1078-0432.Ccr-14-0485.

31.	Stein U, Arlt F, Walther W, Smith J, Waldman T, Harris ED, et al. The metastasis-associated gene S100A4 is a novel target of beta-catenin/T-cell factor signaling in colon cancer. Gastroenterology. 2006;131(5):1486-500. Epub 2006/11/15. doi: 10.1053/j.gastro.2006.08.041.

32.	Horiuchi A, Hayashi T, Kikuchi N, Hayashi A, Fuseya C, Shiozawa T, et al. Hypoxia upregulates ovarian cancer invasiveness via the binding of HIF-1α to a hypoxia-induced, methylation-free hypoxia response element of S100A4 gene. Int J Cancer. 2012;131(8):1755-67. Epub 2012/01/31. doi: 10.1002/ijc.27448.

43.	Liu L, Qi L, Knifley T, Piecoro DW, Rychahou P, Liu J, et al. S100A4 alters metabolism and promotes invasion of lung cancer cells by up-regulating mitochondrial complex I protein NDUFS2. J Biol Chem. 2019;294(18):7516-27. Epub 2019/03/20. doi: 10.1074/jbc.RA118.004365.

44.	Bettum IJ, Gorad SS, Barkovskaya A, Pettersen S, Moestue SA, Vasiliauskaite K, et al. Metabolic reprogramming supports the invasive phenotype in malignant melanoma. Cancer Lett. 2015;366(1):71-83. Epub 2015/06/23. doi: 10.1016/j.canlet.2015.06.006.

45.	Huang J, Zhou B, Wang L, Huang Q, Lin X, Zhao H, et al. S100A4 triggeres the pyroptosis of vsmcs: association with mitochondrial damage, impaired mitophagy, and Ca(2+) dysregulation. Biol Direct. 2026;21(1):18. Epub 2026/01/13. doi: 10.1186/s13062-025-00723-x.
